# Commentary: The embodied brain: towards a radical embodied cognitive neuroscience

**DOI:** 10.3389/fnhum.2015.00669

**Published:** 2015-12-21

**Authors:** Jakub R. Matyja, Krzysztof Dolega

**Affiliations:** ^1^Institute of Philosophy and Sociology, Polish Academy of SciencesWarsaw, Poland; ^2^Institut für Philosophie II, Ruhr-Universität BochumBochum, Germany

**Keywords:** embodied cognition, mechanistic explanations, radical embodied cognitive neuroscience, neurosciences, enactive cognition, explanation in psychology, neuroscience, explanation in neuroscience

Recently, a new paradigm has emerged in mind and brain sciences. Radical embodied neuroscience (REN) aims to respond to the problems of mapping particular cognitive functions to narrowly defined brain regions. Accordingly, the proponents of this approach call for research to move beyond heuristics of localization and decomposition (Bechtel and Richardson, 1993). In this commentary, we focus solely on the functional connectivist blend of REN. Supporters of this position (see e.g., Kiverstein and Miller, 2015 for an interesting analysis of recent works on emotions and cognition) build their argument on the supposed failure of the project to divide the brain into functionally distinct areas responsible for particular cognitive processes. Drawing upon the work on functional connectivism (Anderson, 2010, 2014), they suggest a paradigm shift in brain research from neuroscience focusing on how the brain implements narrowly defined cognitive functions, to one in which the locus of explanation is determined by the dynamic interactions between the brain and the non-neural body embedded in the organism's ecological niche.

We aim to point to two core challenges facing the line of argumentation adopted by the functional connectivist supporters of REN. Firstly, the “how” challenge concerns the lack of guidelines regarding how embodied cognitive neuroscience should proceed and build its explanations without reference to localizable neural underpinnings. This challenge is obviously directed at the more general proposal of a shift toward an embodied understanding of neuroscience. Secondly, the “why” challenge is concerned with the motivation for abandoning (or considering whether to abandon) localization and decomposition, given that current neuroscientific methods of analysis (e.g., network analysis) have meaningfully repurposed these heuristics by drawing on the insights of functional connectivity (Klein, 2012). Finally, we propose that both of these challenges are dissolved by the application of a mechanistic explanation of these phenomena, which not only provides a naturalistically plausible framework (e.g., Miłkowski, 2013; Matyja, 2015) for embodied cognitive neuroscience, but does justice to the work of Anderson (2014) and the opponents of the strong modularity thesis (e.g., Mundale, 2002; Price and Friston, 2005).

## The “how” challenge: isn't it better just to recompose?

REN's call (Kiverstein and Miller, 2015) for reconsideration of the localization (of the neural underpinnings of assumed cognitive function) and decomposition (of particular cognitive processes into explanatorily “manageable” parts) is not without problems. How should neuroscience proceed without these methodological tools? Kiverstein and Miller's positive proposal, which focuses on interpreting contemporary findings on emotion and cognition, does not answer this important question, but is instead based on what we call “that” claims (e.g., “*that*” the localization and decomposition practices should be abandoned in favor of the novel research framework; “*that*” these two research heuristics essentially fail, and “that” it impossible to localize given neural underpinnings—see the discussion below).

Our positive proposal is to answer the “how” challenge by adopting the methodology of the mechanistic explanation framework—the idea that localization and decomposition heuristics are useful to the extent to which they enable researchers to later causally recompose a given cognitive function and its neural correlates into the context of the workings of the organism as a whole. Such recomposition should proceed with the identification of cognitive mechanisms responsible for the occurrence of a given cognitive phenomenon, as well as the identification of the overall role that such mechanisms serve within the context (Bechtel, 2009) of an entire organism and its interactions with environment. These two essential steps seem to be impossible if we abandon the localization and decomposition heuristics altogether, since decomposing of a mechanism requires identifying what its parts are. Moreover, it remains unclear why neuroscience should move beyond these tools, given that new methods, such as network-based analysis, apply exactly those heuristics in order to elucidate the relationship between the neural and extra-neural contexts of the cognitive mechanism and the organism.

## The “why” challenge: why should we go radical?

One of the main reasons proponents of REN question the heuristics of localization and decomposition is the growing evidence (Price and Friston, 2005) against the possibility of mapping singular cognitive functions onto narrowly circumscribed brain regions. The discovery of pluripotency (i.e., the participation of a particular region in carrying out more than one cognitive function, Anderson, 2010, 2014) and degeneracy (i.e., different regions “taking over” the performance of a particular function after a given brain area is damaged or disabled, Friston and Price, 2003; Edelman and Gally, 2001; Figdor, 2010) has led to a debate about individuation of psychological processes which ought to play an explanatory role in neuroscientific research. Such debates about the cognitive ontology of neuroscience have been used by REN supporters to argue that the impossibility of “strict” localization, understood as a one-one mapping of functions to regions, can only be circumvented by using the extra-neural contexts of body and environment to define and individuate psychological functions.

However, in doing this, the REN proponents seem to ignore (with a notable exception of Silberstein and Chemero, 2013) the more advanced method of network analysis. Unlike region-based analysis, the network-based project does not rely on the strict understanding of localization and is compatible with the hypothesis of massive neural redeployment (or MRH) (Anderson, 2007, 2010), according to which brain regions with particular causal roles can be constitutive in different cognitive processes. Network-based analysis respects this insight by allowing for a particular brain region to realize different cognitive functions in virtue of participating within different functional networks. Crucially, such neural contexts can be individuated by testing hypotheses regarding similar (or dissimilar) experimental tasks. Although, individuating the causal role that a single region plays in different functional networks on the basis of task design may sometimes be problematic, task oriented heuristics provide a fertile ground for hypothesis testing and comparison (Klein, 2012), due to clearly defined and controllable parameters. This poses particular problems for the REN supporters. Firstly, it means that although cognitive neuroscience is not committed to a strict notion of localization, it nevertheless remains committed to some form of localization (e.g., non-strict localization in the sense of Anderson's works). Accordingly, it remains unclear why the discipline should follow REN proponents' call to consider extra-neural contexts. Secondly, given the aforementioned compatibility of the network project not only with pluripotency/degeneracy and MRH, but also with localization and recomposition, it is unclear why neuroscience should move beyond examining the relation between neural contexts and tasks. More importantly, why should a definition of cognitive functions in terms of loosely defined ecological contexts be preferred over an identification through clearly delineated and comparable tasks?

## Funding

JM's involvement in this project was funded by National Science Center (Poland) research grant under the decision DEC-2014/14/E/HS1/00803. KD's work is funded by the Volkswagen Foundation research grant “Situated Cognition. Perceiving the world and understanding other minds.” held by Prof. Tobias Schlicht.

### Conflict of interest statement

The authors declare that the research was conducted in the absence of any commercial or financial relationships that could be construed as a potential conflict of interest.
